# Generating Datasets for Anomaly-Based Intrusion Detection Systems in IoT and Industrial IoT Networks

**DOI:** 10.3390/s21041528

**Published:** 2021-02-23

**Authors:** Ismael Essop, José C. Ribeiro, Maria Papaioannou, Georgios Zachos, Georgios Mantas, Jonathan Rodriguez

**Affiliations:** 1 Faculty of Engineering and Science, University of Greenwich, Chatham Maritime ME4 4TB, UK; i.a.essop@gre.ac.uk (I.E.); m.papaioannou@av.it.pt (M.P.); g.zachos@gre.ac.uk (G.Z.); gimantas@av.it.pt (G.M.); 2 Instituto de Telecomunicações, Aveiro 3810-193, Portugal; jonathan@av.it.pt; 3 Faculty of Computing, Engineering and Science, University of South Wales, Pontypridd CF37 1DL, UK

**Keywords:** IoT, Industrial IoT, benign datasets generation, malicious datasets generation, Cooja simulator, Contiki OS, anomaly-based intrusion detection

## Abstract

Over the past few years, we have witnessed the emergence of Internet of Things (IoT) and Industrial IoT networks that bring significant benefits to citizens, society, and industry. However, their heterogeneous and resource-constrained nature makes them vulnerable to a wide range of threats. Therefore, there is an urgent need for novel security mechanisms such as accurate and efficient anomaly-based intrusion detection systems (AIDSs) to be developed before these networks reach their full potential. Nevertheless, there is a lack of up-to-date, representative, and well-structured IoT/IIoT-specific datasets which are publicly available and constitute benchmark datasets for training and evaluating machine learning models used in AIDSs for IoT/IIoT networks. Contribution to filling this research gap is the main target of our recent research work and thus, we focus on the generation of new labelled IoT/IIoT-specific datasets by utilising the Cooja simulator. To the best of our knowledge, this is the first time that the Cooja simulator is used, in a systematic way, to generate comprehensive IoT/IIoT datasets. In this paper, we present the approach that we followed to generate an initial set of benign and malicious IoT/IIoT datasets. The generated IIoT-specific information was captured from the Contiki plugin “powertrace” and the Cooja tool “Radio messages”.

## 1. Introduction

Despite the significant benefits that IoT and Industrial IoT (IIoT) networks bring to citizens, society, and industry, the fact that these networks incorporate a wide range of different communication technologies (e.g., WLANs, Bluetooth, and Zigbee) and types of nodes/devices (e.g., temperature/humidity sensors), which are vulnerable to various types of security threats, raises many security and privacy challenges in IoT/IIoT-based systems. For instance, attackers may compromise IoT/IIoT networks in order to manipulate sensing data (e.g., by injecting fake data) and cause malfunction to the IoT/IIoT-based systems that rely on the compromised IoT/IIoT networks. It is worthwhile to mention that IoT/IIoT networks can become an attractive target of attackers with a wide spectrum of motivations ranging from criminal intents aimed at financial gain to industrial espionage and cyber-sabotage. Therefore, security solutions protecting IoT/IIoT networks from attackers are critical for the acceptance and wide adoption of such networks in the coming next years. Nevertheless, the high resource requirements of complex and heavyweight conventional security mechanisms cannot be afforded by (i) the resource-constrained IoT/IIoT nodes (e.g., sensors) with limited processing power, storage capacity, and battery life; and/or (ii) the constrained environment in which the nodes are deployed and interconnected using lightweight communication protocols. Consequently, there is an urgent need for novel security mechanisms, such as accurate and efficient anomaly-based intrusion detection systems (AIDSs) tailored to the resource-constrained characteristics of IoT/IIoT networks, to be developed in order to address the pressing security challenges of IoT/IIoT networks with reasonable cost, in terms of processing and energy, before IoT/IIoT networks gain the trust of all involved stakeholders and reach their full potential in the market [1,2,3]. However, there is a lack of up-to-date, representative and well-structured IoT/IIoT-specific datasets that are publicly available to the research community and constitute benchmark datasets for training and evaluating machine learning (ML) models used in AIDSs for IoT/IIoT networks [4,5]. This lack of benchmark IoT/IIoT datasets constitutes a significant research gap that should be addressed in order to develop more accurate and efficient IoT/IIoT-specific AIDS whose effectiveness is evaluated based on their performance to detect IoT/IIoT attacks which is a process reliant on comprehensive IoT/IIoT-specific datasets.

In fact, although several datasets, such as KDDCUP99 [6], NSL-KDD [7], UNSW-NB15 [8], and CICD2017 [9] have been created over the past two decades for evaluation purposes of network-based intrusion detection systems (IDSs), they do not include any specific characteristics of IoT/IIoT networks as these datasets do not contain sensors’ reading data or IoT/IIoT network traffic [4,5]. To respond to this major issue, few efforts focused on the generation of IoT-specific datasets have also been seen in the literature recently. However, they are characterised by some limitations in terms of the IoT-specific information they include. For instance, the datasets proposed in [10,11] are IoT-specific datasets but they lack of events reflecting attack scenarios. To address this limitation, the IoT-specific and network-related datasets proposed in [12,13] contain events reflecting attack scenarios; however, they do not cover a diverse set of attack scenarios and do not include sensors’ reading data or information related to the behaviour of the IoT/IIoT devices (e.g., sensors/actuators) within the network. Therefore, these IoT datasets can mainly be used for detecting only a limited number of network-based attacks against IoT/IIoT networks as they do not contain adequate information for detecting a wide range of network-based attacks and/or attacks that manipulate sensor measurement data or compromise IoT/IIoT devices within the IoT/IIoT network.

Consequently, there is an urgent need for comprehensive IoT/IIoT-specific datasets containing not only network-related information (e.g., packet-level information and flow-level information) but also events reflecting multiple benign and attack scenarios from current IoT/IIoT network environments, sensor measurement data, and information related to the behaviour of the IoT/IIoT devices deployed within the IoT/IIoT network for efficient and effective training and evaluation of AIDSs suitable for IoT/IIoT networks. Towards this direction, the recent work of [4] has proposed, for the first time, to the best of our knowledge, a new dataset that includes events of a variety of IoT-related attacks and legitimate scenarios, IoT telemetry data collected from heterogeneous IoT/IIoT data sources, network traffic of IoT/IIoT network, and audit traces of operating systems [4]. Therefore, it is clear that more comprehensive IoT/IIoT-specific datasets including events reflecting multiple benign and attack scenarios, sensor measurement data, network-related information, and information related to the behaviour of the IoT/IIoT devices are required to be generated and become publicly available to the research community so as to fill this significant research gap of lack of benchmark IoT/IIoT datasets and more accurate and efficient IoT/IIoT-specific AIDS to be developed.

Contribution to filling this research gap is the main target of our recent research work. In particular, our focus is on the generation of new labelled IoT/IIoT datasets that will be publicly available to the research community and include: (a) events reflecting multiple benign and attack scenarios from current IoT/IIoT network environments, (b) sensor measurement data, (c) network-related information (e.g., packet-level information and flow-level information) from the IoT/IIoT network, and (d) information related to the behaviour of the IoT/IIoT devices deployed within the IoT/IIoT network. It is worthwhile to mention that the new labelled IoT/IIoT datasets are generated by implementing various benign IoT/IIoT network scenarios and IoT/IIoT network attack scenarios in the Cooja simulator which is the companion network simulator of the open source Contiki Operating System (OS) that is one of the most popular OSs for resource constrained IoT devices [14]. To the best of our knowledge, this is the first time that the Cooja simulator is going to be used, in a systematic way, to generate comprehensive IoT/IIoT datasets. In this paper, we present the approach that we followed to generate an initial set of benign IoT/IIoT datasets (i.e., including only normal events) and malicious IoT/IIoT datasets (i.e., including attack and normal events) by utilising the Cooja simulator that was the simulation environment where the corresponding benign and attack scenarios were implemented.

The rest of this paper is organised as follows. In Section 2, the main threats against the IoT/IIoT network (i.e., perception domain) are presented and in Section 3, examples of anomaly-based intrusion detection systems for IoT/IIoT networks are discussed. In Section 4, a detailed description of the approach followed to generate a set of benign datasets by implementing a benign IIoT network scenario in the Cooja simulator is provided. In Section 5, a detailed description of the approach followed to generate a set of malicious datasets by implementing a User Datagram Protocol (UDP) flooding attack scenario in the Cooja is provided as well. In Section 6, a discussion on the generated datasets is given. Finally, Section 7 concludes this paper. 

## 2. Threat Analysis of the IoT/IIoT Network (Perception Domain)

**Figure 1:**
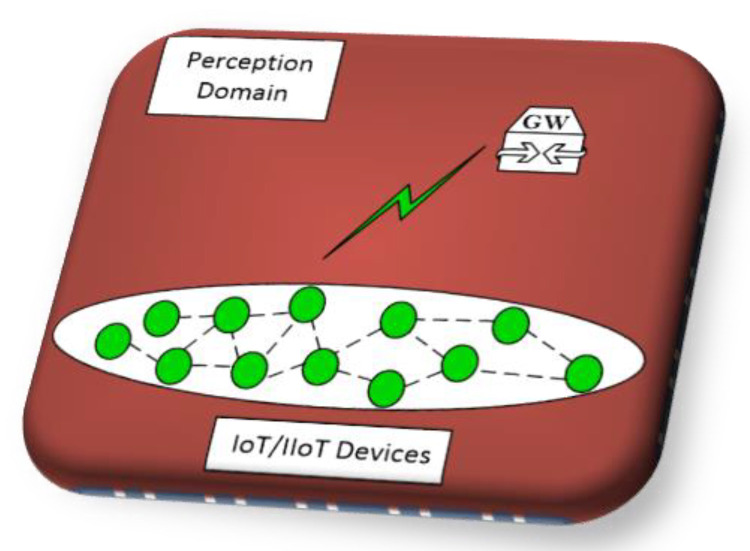
IoT/IIoT Network (Perception Domain).

The perception domain, as shown in Figure 1, can be perceived as the device layer in the ITU-T reference model [15]. As the main purpose of the perception domain is to gather data, the security challenges in this domain target to forge collected IoT/IIoT data and damage perception devices, as presented below.

### 2.1. Sinkhole Attacks

In this type of attacks, a compromised IoT/IIoT node (i.e., IoT/IIoT gateway [16]) in the perception domain proclaims very appealing capabilities of power, computation and communication [17] so that nearby nodes (i.e., IoT/IIoT sensors) will choose it as the forwarding node in the routing process due to its very attractive capabilities. As a consequence, the compromised IoT/IIoT node can increase the amount of data obtained before it is delivered to the cloud domain of the IoT-based monitoring system. Therefore, a sinkhole attack can not only compromise the confidentiality of the manufacturing data but also can comprise an initial step to launch additional attacks such as DoS/DDoS attacks [17,18].

### 2.2. Node Capture Attacks

In this type of attack, the adversary is able to extract important information about the captured node, such as the group communication key, radio key, etc. [17]. Additionally, the adversary can copy the important information related to the captured node to a malicious node, and afterwards fake the malicious node as a legitimate node to connect to the IoT/IIoT network (i.e., perception domain). This type of attack is also known as node cloning/replication attack [17,19]. This attack may lead to compromising the security of the complete IoT/IIoT-based monitoring system. 

### 2.3. Malicious Code Injection Attacks

An attacker can take control of an IoT/IIoT node or device in the perception domain by exploiting its security vulnerabilities in software and hardware and injecting malicious code into its memory. Afterwards, using the malicious code, the attacker can force the node or device to perform unintended operations. For example, the infected IoT/IIoT node(s) or device(s) can be used as a bot(s) to launch further attacks (e.g., DoS and DDoS) against other devices or nodes within the perception domain or even against the other domains (i.e., Network domain and Cloud domain). In addition, the attacker can use the injected malicious code in the infected device or node to get access into the IoT/IIoT-based system and/or get full control of the system [19].

### 2.4. False Data Injection Attacks

After capturing an IoT/IIoT node or device in the perception domain, the adversary can inject false data in place of benign data measured by the captured IoT/IIoT node or device and transmit the false data to the Cloud domain [17]. Thereafter, receiving the false data, the IoT/IIoT-based system may provide wrong services, which further negatively impacts the effectiveness of system itself. 

### 2.5. Replay Attacks

In the perception domain, the attacker can use a malicious IoT/IIoT node or device to transmit to the destination host (i.e., IoT/IIoT gateway) with legitimate identification information, already received by the destination host, so that the malicious node or device can become a trusted node/device to the destination host [17]. Replay attacks are commonly launched in authentication process to destroy the validity of certification. 

### 2.6. Eavesdropping

As the IoT/IIoT nodes and devices in perception domain communicate via wireless networks, an attacker (i.e., eavesdropper) can retrieve sensitive manufacturing data by overhearing the wireless transmission. For instance, an adversary within the perception domain can eavesdrop exchanged information by tracking wireless communications and reading the contents of the transmitted packages [17]. The eavesdropper can passively intercept the wireless communication between a sensor (e.g., environment industrial sensors or sensors on the machine resources) and the IoT/IIoT gateway, and extract confidential data (e.g., through traffic analysis) in order to maliciously use them. 

### 2.7. Sleep Deprivation Attacks or Denial of Sleep Attacks

These attacks target to drain the battery of the resource constrained IoT/IIoT devices of the perception domain. In principle, the IoT/IIoT devices in the perception domain are usually programmed to follow a sleep routine when they are inactive in order to reduce the power consumption and extend their life cycle. However, an adversary may break the programmed sleep routines and keep the IoT/IIoT devices of the perception domain continuously active until they are shut down due to a drained battery. Attackers can achieve this by running infinite loops in these devices using malicious code or by artificially increasing their power consumption [20].

### 2.8. Sybil Attacks

In a sybil attack, a malicious or sybil node or device can illegitimately claim multiple identities, allowing it to impersonate them within the perception domain. For instance, the malicious node can achieve to connect with several other devices in order to maximise its influence and even deceive the complete system to draw incorrect conclusions [21].

### 2.9. Denial of Service (DoS) Attacks

The main target of these attacks is to deplete resources of the perception domain in order to make the whole IoT/IIoT network or specific nodes (e.g., machine or/and environment resources) or devices (e.g., IoT/IIoT gateway) unavailable. For instance, jamming attacks are a type of DoS attacks where an attacker transmits a high-range signal to overload the communication channel between two communicating entities and disrupt their communication. Within the perception domain of the IoT/IIoT-based system, jamming attacks can disrupt the communication between the IoT/IIoT sensors and the Gateway in order to prevent data from being transmitted to the Gateway, leading to malfunctions in the provided services to the authorised users. Jamming attacks can be performed by passively listening to the wireless medium so as to broadcast on the same frequency band as the legitimate transmitting signal. Finally, distributed denial of service (DDoS) attacks are a large-scale variant of DoS attacks and in the case of the perception domain an example of DDoS attack is when a large number of nodes (e.g., IoT/IIoT sensors) are compromised so as to flood the Gateway with a lot of transmitted data/requests and render it unavailable or disrupt its normal operations [22,23].

## 3. Anomaly-Based Intrusion Detection Systems for IoT/IIoT Networks

In this Section, two examples of anomaly-based intrusion detection systems for IoT/IIoT networks are discussed. Moustafa et al. in [24] proposed an ensemble network intrusion detection technique which utilises established statistical flow features. The goal is to mitigate malicious events, and more specifically botnet attacks against DNS, HTTP and MQTT protocols that are employed in IoT networks. The first step of their work revolves around the deep analysis of the TCP/IP model and the subsequent extraction of a set of features from the network traffic protocols MQTT, HTTP, and DNS protocols. The Bro-IDS tool is used by the authors for basic features while they also employ, in parallel, their own extractor module to generate additional statistical features of the transactional flows. Consequently, features are filtered and only the most important ones are selected in order to simplify the NIDS and decrease its computational cost. In this step, the authors utilise the correlation coefficient on result features as a means of features selection. Lastly, an AdaBoost ensemble learning method is developed to detect the attacks. The method is based on the combination of three different Machine Learning (ML) algorithms; decision tree (DT), Naive Bayes (NB), and artificial neural network (ANN) algorithms. These classification techniques were chosen mainly due to the core entropy measure that was calculated from the feature vectors. The AdaBoost (Adaptive Boosting) method improves the performance of the detection in comparison to using each machine learning algorithm separately. In case of small differences of the feature vectors, an error function is employed. The importance of the error function lies in computing the error value for each instance of the distributed input data. Based on this error value, it is possible to understand and evaluate which learners are best suited to classify each instance. The experiments results show that the ensemble technique achieved a high detection rate (95.25%–99.86%) and a low false positive rate (between 0.01% and 0.72%) compared to existing state-of-the-art techniques. The authors employed the UNSWNB15 and NIMS botnet datasets with simulated IoT sensor data to support their findings.

Furthermore, a multi-layer perceptron (MLP), which is a type of supervised artificial neural network [25]), is used in an offline IDS for IoT networks [26]. The ANN consists of 3 layers and each of the hidden and output layers’ neurons use a unipolar sigmoid transfer function to transform their input values to a specific output value. The network was trained using a stochastic learning algorithm with mean square error function. The training process included both feed-forward and backward training algorithms. To perform its task, the ANN analyses the Internet packet traces and attempts to detect DoS and DDoS attacks in IoT network. In order to evaluate the IoT IDS, an experimental architecture was created with four client nodes and a server relay node. The server node was subjected not only to DOS attacks from a single host with more than 10 million UDP packets sent but also to DDoS attacks from three hosts each sending over 10 million UDP packets at wire speed. The results of their simulations showed a detection accuracy of 99.4% and 0.6% false positive rate. The authors used a training dataset consisting of a total of 2313 samples, 496 of them deployed for validation and 496 of them for testing [5].

## 4. Generation of Benign IoT/IIoT Datasets

**Figure 2:**
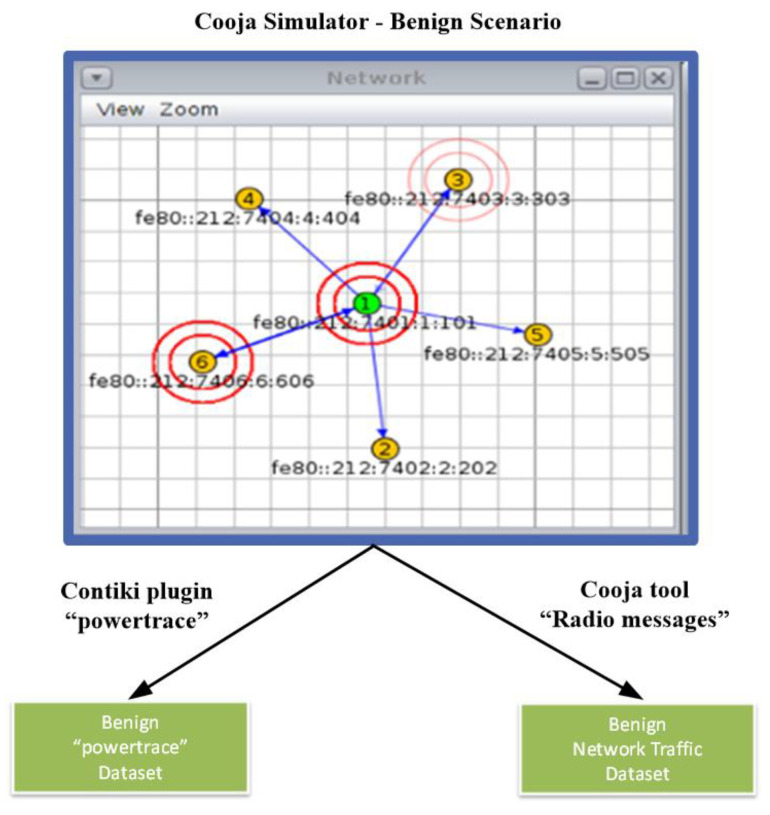
Benign datasets generation by utilizing the Cooja simulator.

In this Section, we provide a detailed description of the approach followed to generate a set of benign datasets by implementing a benign IoT/IIoT network scenario in the Cooja simulator, as shown in Figure 2. The generated IoT/IIoT-specific information from the simulated scenario was captured from the Contiki plugin “powertrace” (i.e., features such as CPU consumption) and the Cooja tool “Radio messages” (i.e., network traffic features) in order to generate the “powertrace” dataset and the network traffic dataset for the simulated benign IoT/IIoT network scenario.

**Figure 3:**
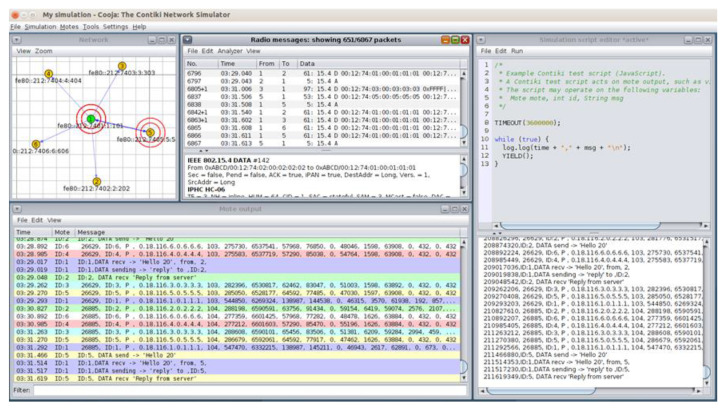
Cooja Simulator—motes’ outputs.

The network topology of the simulated benign IoT/IIoT network scenario in the Cooja simulator environment consists of 5 yellow UDP-client motes (i.e., motes 2, 3, 4, 5 and 6) and the green UDP-server mote (i.e., mote 1), as depicted in Figure 2. The simulation duration was set to 60 min and the motes’ outputs were printed out in the respective window (e.g., Mote output) while simulations run, as shown in Figure 3. In addition, the yellow UDP-client motes were configured to send text messages every 10 s, approximately, to the green UDP-sever mote that was configured to provide a corresponding response. The UDP protocol was used at the Transport Layer and the IPv6 at the network layer. Moreover, the type of motes used in this scenario was the Tmote Sky that is an ultra-low power wireless module for use in sensor networks, monitoring applications, and rapid application prototyping. In addition, Tmote Sky motes leverage industry standards such as USB and IEEE 802.15.4 to interoperate seamlessly with other devices. By using industry standards, integrating humidity, temperature, and light sensors, and providing flexible interconnection with peripherals, Tmote Sky motes enable several mesh network applications [27]. 

### 4.1. Benign “Powertrace” Dataset Generation

#### 4.1.1. Benign “Powertrace” Dataset Generation

**Figure 4:**
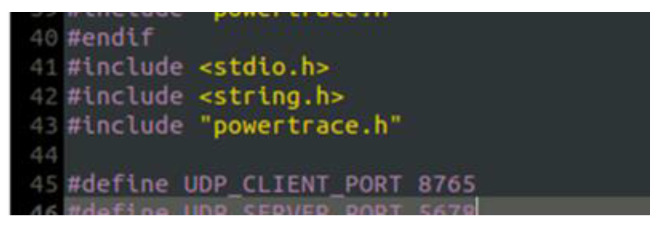
“powertrace.h” library in the mote code.

**Figure 5:**
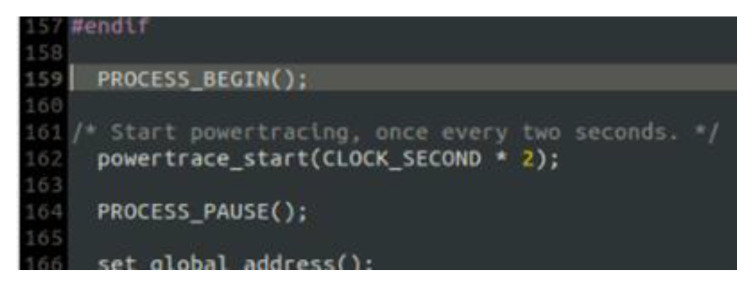
Powertracing Begin.

The “powertrace” dataset includes information about features such as total CPU energy consumption and low power mode (LPM) energy consumption. In fact, it is the dataset of the simulated benign IIoT network scenario that includes records about information related to the energy consumption of the IIoT devices (i.e., motes) deployed within the simulated IIoT network. To enable the “powertrace” plugin and generate the “powertrace” dataset, we programmed the motes of the benign IIoT network to make use of the “powertrace” plugin for collecting “powertrace” related features every 2 s. In particular, we included the “powertrace.h” library into the code of each mote (i.e., #include “powertrace.h”), as shown in Figure 4, and defined to start powertracing, once every 2 s, in the code of each mote as shown in Figure 5.

More precisely, the “powertrace” plugin captured raw information, every 2 s, about the set of features summarised in Table 1. In particular, the “powertrace” plugin tracks the duration (i.e., number of cpu ticks) of activities of a mote being in each power state. Particularly, the outputs demonstrate the fraction of time in which a mote remains for a given power state. There are the following six power states: (i) cpu; (ii) lpm; (iii) transmit; (iv) listen; (v) idle_transmit; and (vi) idle_listen, as shown in Table 1. These are measured with a hardware timer (i.e., clock frequency is defined in RTIMER_SECOND or 32,768 Hz for XM1000).

**Figure 6:**
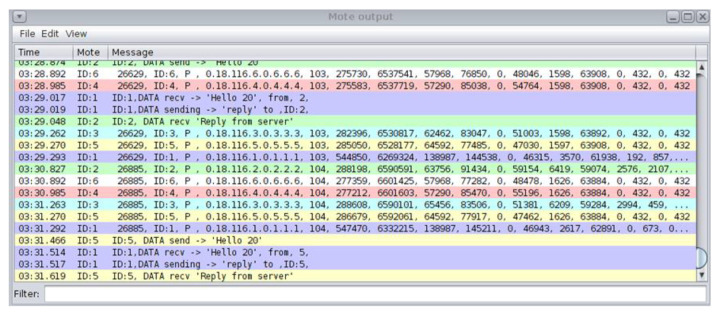
Cooja Simulator—Mote output window.

In Figure 6, the depicted Mote output window displays the captured “powertrace” information every 2 s and also the messages sent and received by each mote (printouts/printf messages from each mote).

**Figure 7:**
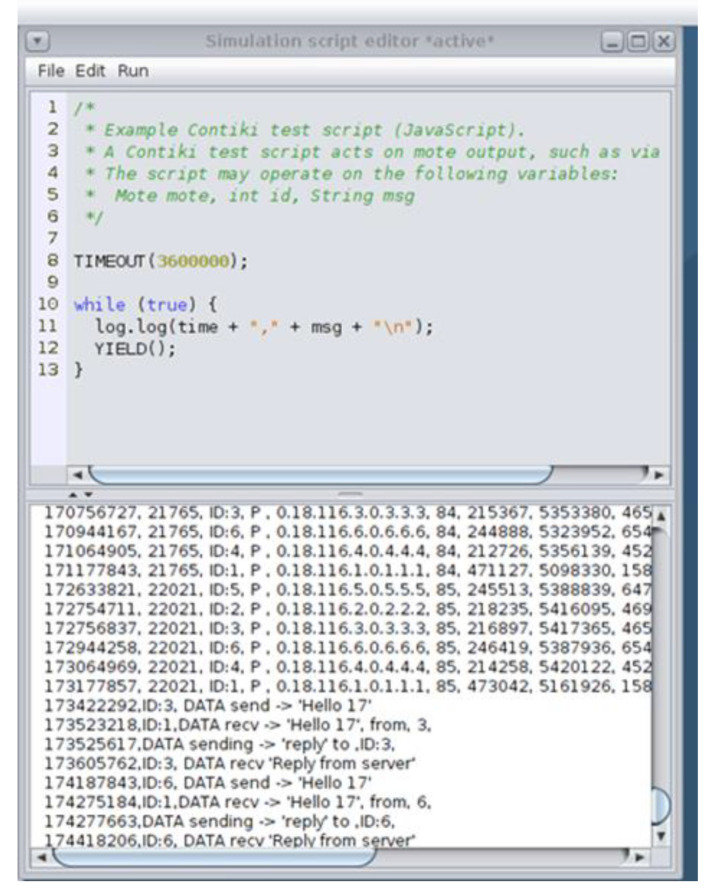
Simulation script editor.

Furthermore, the Simulation script editor, shown in Figure 7, is a Cooja tool used to display messages and set a timer on the simulation. As shown in Figure 7, the upper part of the Simulation script editor was used to create scripts and the lower part to show the captured “powertrace” information and the printouts (i.e., printf messages) from the motes until the timeout occurs. In our implementation, we considered the simulation duration to be 60 min and thus, the timeout was set at 3,600,000 ms. When the timeout occurred, the simulation stopped, and all the captured information and prints were stored in the log file named “COOJA.testlog”.

**Figure 8:**
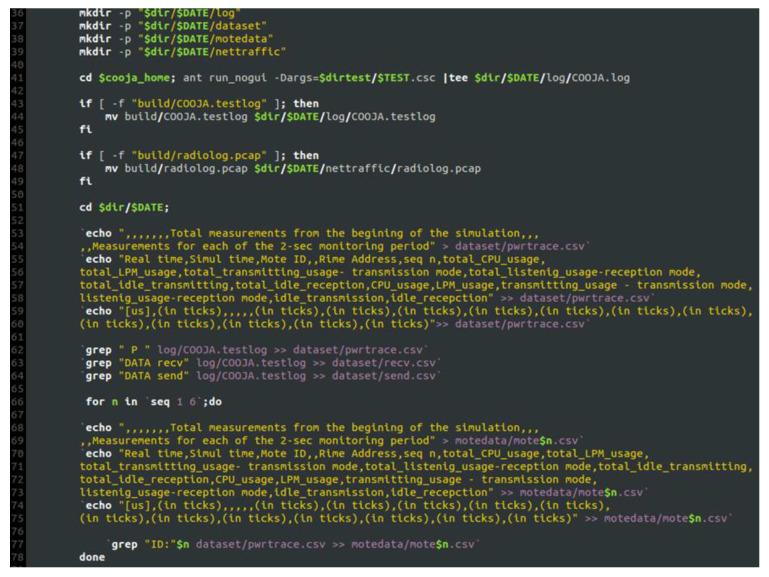
Extract of the “IoT_Simul.sh” bash file.

Having collected all the captured raw information from the “powertrace” plugin in the “COOJA.testlog” file, the challenging task was to extract this information from the “COOJA.testlog” file to a csv file that would be the “powertrace” dataset of the simulated benign IIoT network scenario including records about the energy consumption of the motes. To address this challenge, we developed the “IoT_Simul.sh” bash file in order to extract all the required “powertrace” information from the “COOJA.testlog” file to the “pwrtrace.csv” file. An extract of the “IoT_Simul.sh” bash file is shown in Figure 8.

**Figure 9:**
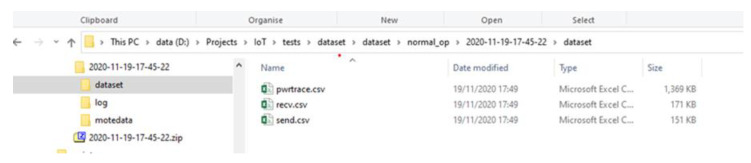
Location of the generated “pwrtrace.csv”, “recv.csv”, and “send.csv” files by the “IoT_Simul.sh” file.

Initially, the “IoT_Simul.sh” file created the root folder which was named with the simulation date and time (i.e., “2020-11-19-17-45-22” folder), as shown below in the left part of Figure 9. Afterwards, the bash file created the “log” folder, inside the “2020-11-19-17-45-22” folder, where the “COOJA.testlog” file was copied from the “…/cooja/build” folder located in the Cooja Simulator environment.

In addition, in the “IoT_Simul.sh” file, we used the Linux tool “grep” in order to extract the required “powertrace” information by selecting the label “P” in each powertrace row (i.e., grep “P” log/COOJA.testlog >> dataset/pwrtrace.csv) from the “COOJA.testlog” file and save it in the “pwrtrace.csv” file in the “dataset” folder that was created by the batch file inside the “2020-11-19-17-45-22” folder, as shown in the left part of Figure 9. In the “dataset” folder, apart from the “pwrtrace.csv” file, the “IoT_Simul.sh” file generated two more files, based on the information included in the “COOJA.testlog” file, as shown in Figure 9; the “recv.csv” file and the “send.csv” file that include the “received” and “sent”messages printed by the motes, respectively. 

Finally, the “IoT_Simul.sh” file extracted the information related to each mote, from the “pwrtrace.csv” file, and generated one csv file for each mote with the corresponding information from the “pwrtrace.csv” file. The generated 6 csv files (i.e., mote1.csv, mote2.csv, mote3.csv, mote4.csv, mote5.csv, mote6.csv) were stored in the “motedata” folder. The “motedata” folder was also created by the “IoT_Simul.sh” file inside the “2020-11-19-17-45-22” folder.

**Figure 10:**
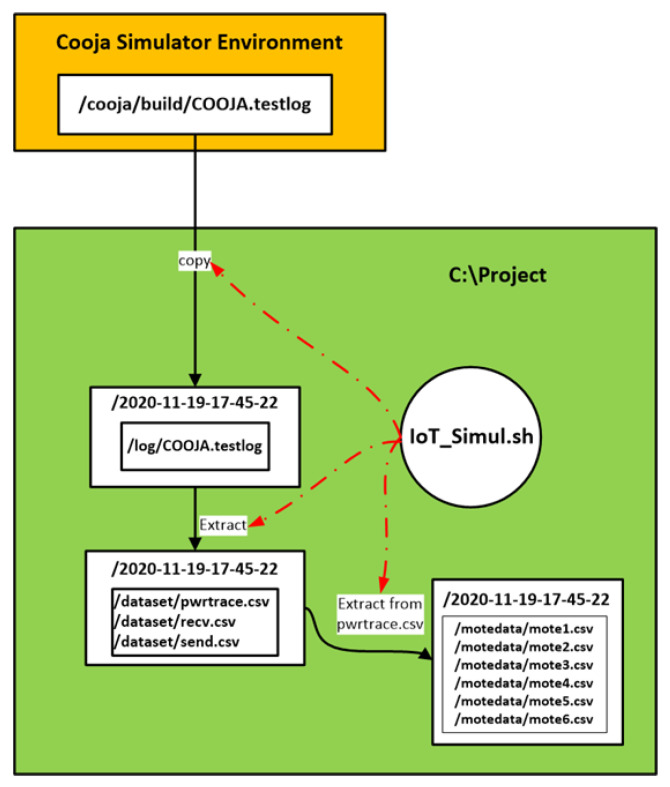
An overview of the process followed by the “IoT_Simul.sh” file to extract all the required “powertrace” information from the “COOJA.testlog” file.

An overview of the above mentioned process followed to extract the required information from the “COOJA.testlog” file to the “pwrtrace.csv”, “recv.csv”, and “send.csv”, “mote1.csv”, “mote2.csv”, “mote3.csv”, “mote4.csv”, “mote5.csv”, and “mote6.csv” files are depicted in the Figure 10.

#### 4.1.2. Benign “Powertrace” Datasets—Results

**Figure 11:**
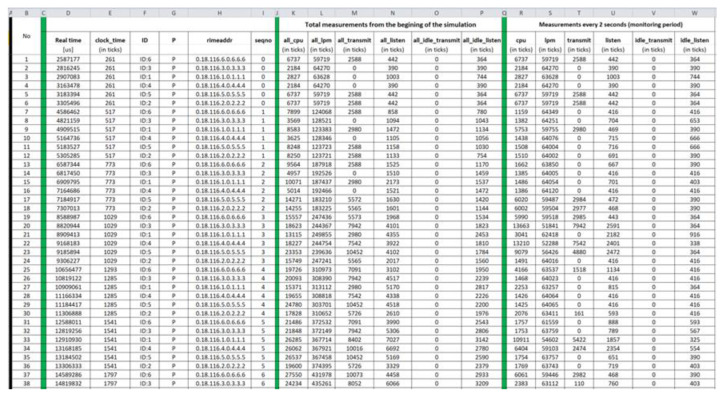
Benign “pwrtrace.csv”—1 to 38 records.

**Figure 12:**
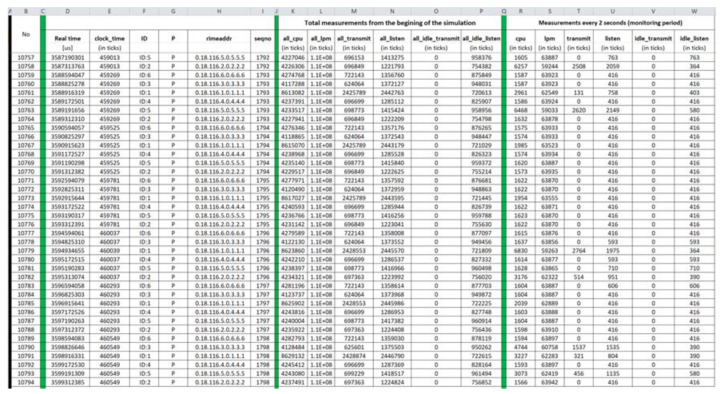
Benign “pwrtrace.csv”—10,757 to 10,794 records.

Benign “pwrtrace.csv”: The generated benign “pwrtrace.csv” file consists of 10,794 records and its first 38 records (i.e., 1–38) and its last 38 records (10,757–10,794) are depicted in Figure 11 and Figure 12, respectively.

**Figure 13:**
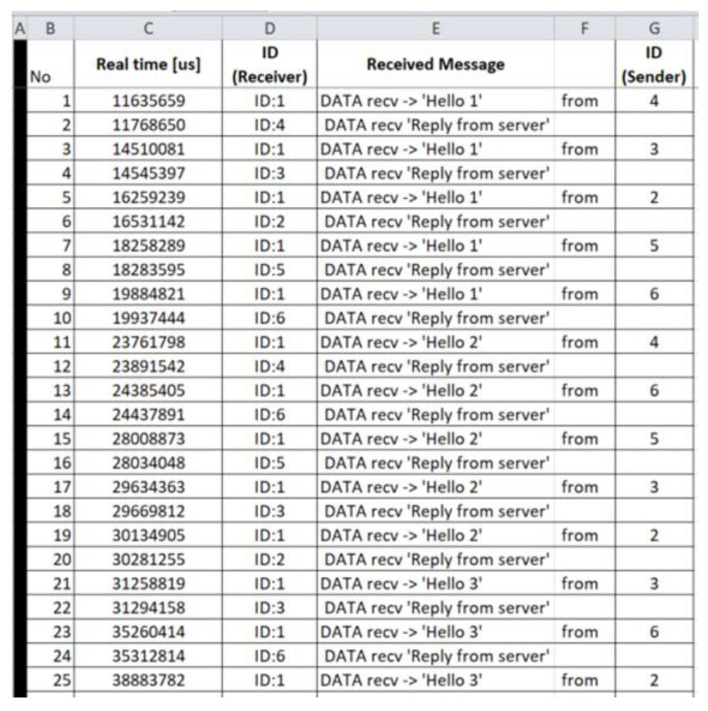
Benign “recv.csv”—1 to 25 records.

Benign “recv.csv”: The generated benign “recv.csv” file consists of 3586 records and its first 25 records (i.e., 1–25) are depicted below in Figure 13.

### 4.2. Benign Network Traffic Dataset Generation

#### 4.2.1. Benign Network Traffic Dataset Generation

**Figure 14:**
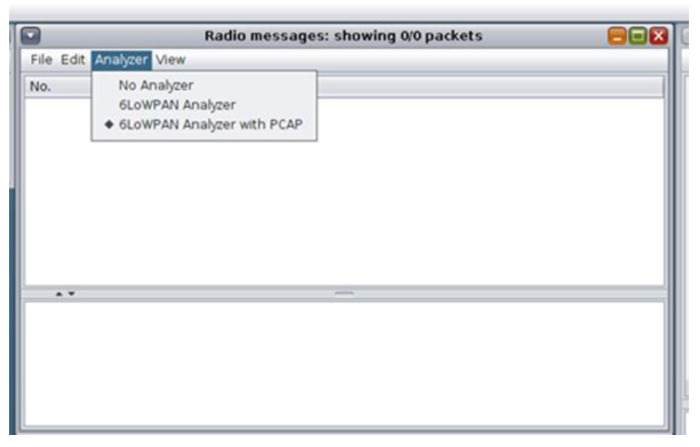
“Radio messages” tool—output window.

The generated network traffic dataset constitutes the dataset of the simulated benign IIoT network scenario that includes records consisting of IIoT network traffic features such as source/destination IPv6 address, packet size, and communication protocol. The Cooja simulator provides the “Radio messages” tool that allowed the collection of data related to the corresponding network traffic features. In Figure 14, the “Radio messages” output window is depicted along with the three configuration options that are provided by the “Radio messages” tool:

The “6LoWPAN Analyzer with PCAP” option was selected and the “Radio messages” tool saved the captured network traffic data from the simulated IIoT network into a pcap file whose file-naming format was as follows: “radiolog-” + System.currentTimeMillis() + “pcap”. 

**Figure 15:**
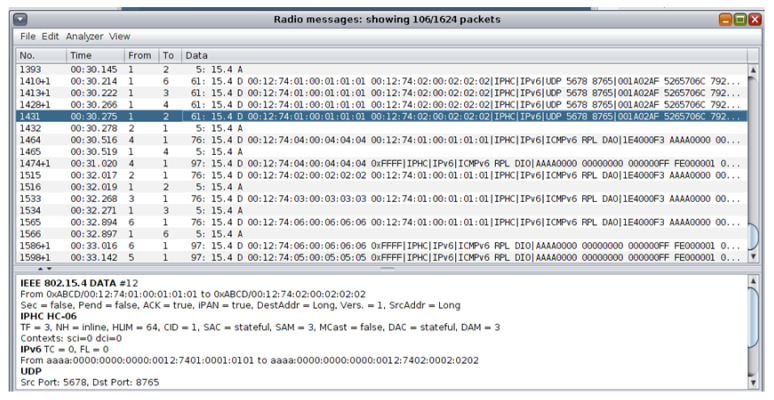
Network traffic information from the benign scenario in the “Radio messages” output window.

During the simulation, the network traffic information about the transmitted data was also being shown in the top part of the “Radio messages” output window as depicted in the top part of Figure 15. When the simulation stopped, the generated pcap file was saved as “radiolog-1605811324302.pcap” within the “…/cooja/build” folder. 

Having now saved all the captured raw network traffic information, through the “Radio messages” tool, into a pcap file, the challenging task was to extract this information from the pcap file to a csv file that would be the network traffic dataset of the simulated benign IIoT network scenario. This challenge was addressed by utilising the “IoT_Simul.sh” file that was also used in the “powertrace” dataset generation process, as described in Section 4.1, and the well-known network protocol analyser Wireshark [28]. 

**Figure 16:**
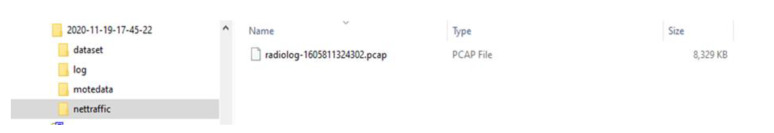
The “nettraffic” folder inside the root folder “2020-11-19-17-45-22” and the copy of the “radiolog-1605811324302.pcap” file.

In particular, the first step was the use of the “IoT_Simul.sh” file in order to copy the “radiolog-1605811324302.pcap” file from the “…/cooja/build” folder located in the Cooja Simulator environment to the “nettraffic” folder that was created by the “IoT_Simul.sh” file inside the root folder “2020-11-19-17-45-22” that was also created by the “IoT_Simul.sh” during the “powertrace” dataset generation process. The “nettraffic” folder inside the root folder “2020-11-19-17-45-22” and the copy of the “radiolog-1605811324302.pcap” file in the “nettraffic” folder is shown in Figure 16.

**Figure 17:**
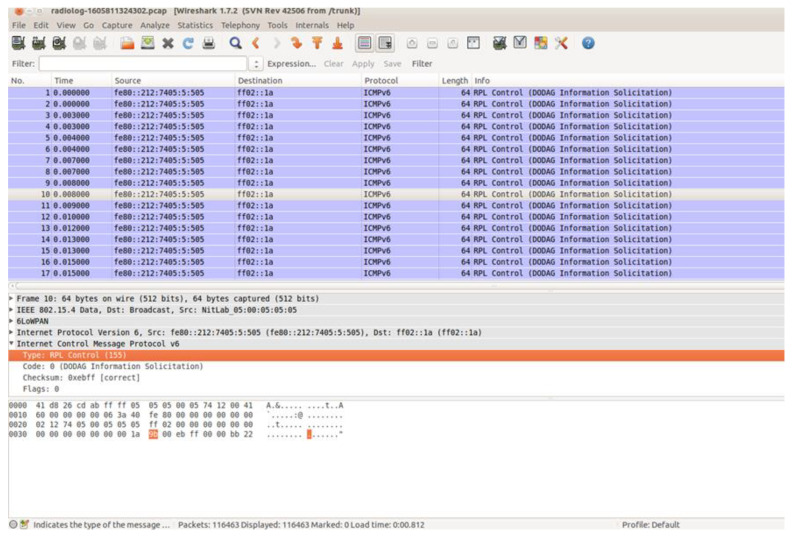
The first seventeenth packets in the “radiolog-1605811324302.pcap” file.

After having the copy of the “radiolog-1605811324302.pcap” file in the “nettraffic” folder, the next step was the extraction of the stored network traffic information from the “radiolog-1605811324302.pcap” file to the “radiolog.csv” file. This was achieved through Wireshark as Wireshark allows opening a pcap file and exporting data to a csv file. In Figure 17, the upper panel of the Wireshark window shows the seventeen first packets included in the “radiolog-1605811324302.pcap” file that was opened via Wireshark. The middle panel shows the protocol details of the 10th packet selected in the upper panel and the bottom panel presents the protocol details of the selected 10th packet in both HEX and ASCII format.

**Figure 18:**
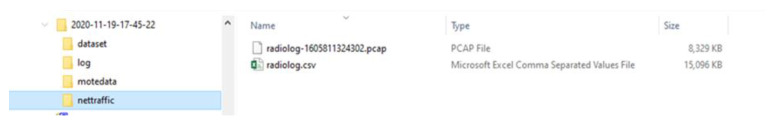
The “radiolog.csv” file in the “nettraffic” folder in the project environment.

**Figure 19:**
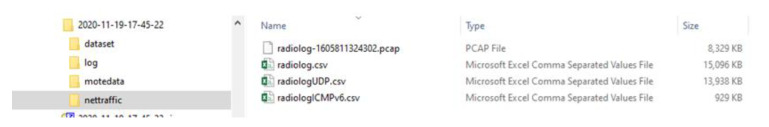
The “radiologICMPv6.csv” file and the “radiologUDP.csv” file in the “nettraffic” folder in the project environment.

The data from the “radiolog-1605811324302.pcap” file were exported and saved, through Wireshark, into the “radiolog.csv” file in the “nettraffic” folder in the project environment, as shown in Figure 18. Furthermore, it is worthwhile to mention that we also used Wireshark to filter the “radiolog-1605811324302.pcap” file based on the ICMPv6 protocol and the UDP protocol and then exported and saved the filtered results, through Wireshark, in the “radiologICMPv6.csv” file and the “radiologUDP.csv” file, respectively, in the “nettraffic” folder in the project environment, as shown in Figure 19. The radiologICMPv6.csv” file and the “radiologUDP.csv” file facilitated the analysis of the capture traffic as shown in Section 6.

**Figure 20:**
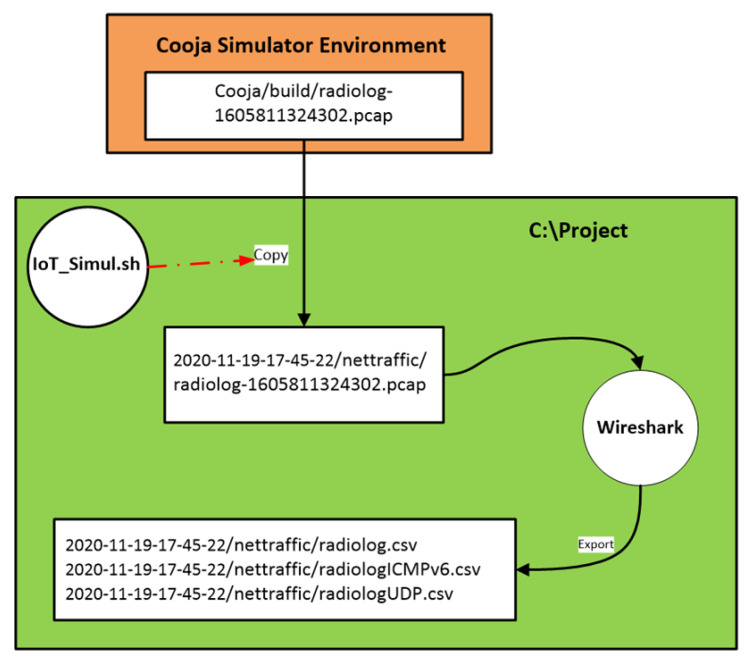
An overview of the process followed to extract all the required network traffic information from the “radiolog-1605811324302.pcap” file.

Finally, an overview of the above mentioned process followed to extract the required information from the “radiolog-1605811324302.pcap” file to the “radiolog.csv”, “radiologICMPv6.csv” and “radiologUDP.csv” files is depicted in Figure 20.

#### 4.2.2. Benign Network Traffic Datasets—Results

**Figure 21:**
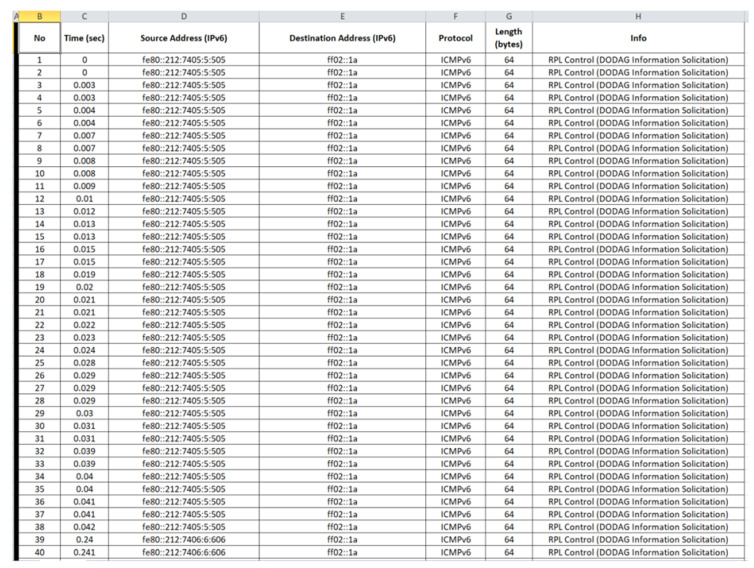
Benign “radiolog.csv”—1 to 40 records.

“radiolog.csv”: The generated benign “radiolog.csv” file consists of 116,463 records and its first 40 records (i.e., 1–40) are depicted below in Figure 21.

**Figure 22:**
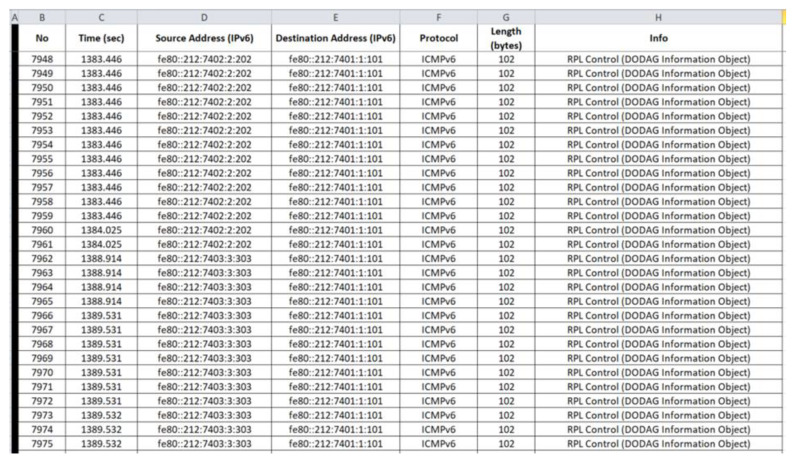
Benign “radiologICMPv6.csv”—7948 to 7975 records.

“radiologICMPv6.csv”: The generated benign “radiologICMPv6.csv” file consists of 7975 records and its last 28 records (i.e., 7948–7975) are depicted below in Figure 22.

**Figure 23:**
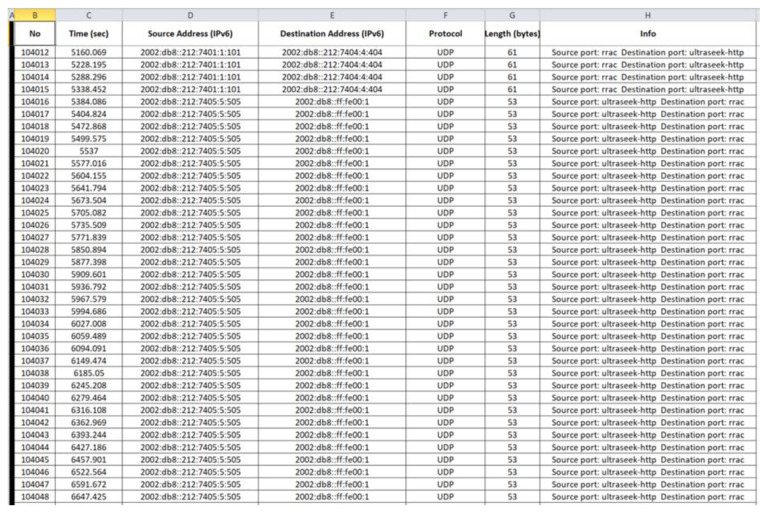
Benign “radiologUDP.csv”—104,012 to 104,048 records.

“radiologUDP.csv”: The generated benign “radiologUDP.csv” file consists of 104,048 records and its last 37 records (i.e., 104,012–104,048) are depicted below in Figure 23.

## 5. Generation of Malicious IoT/IIoT Datasets

**Figure 24:**
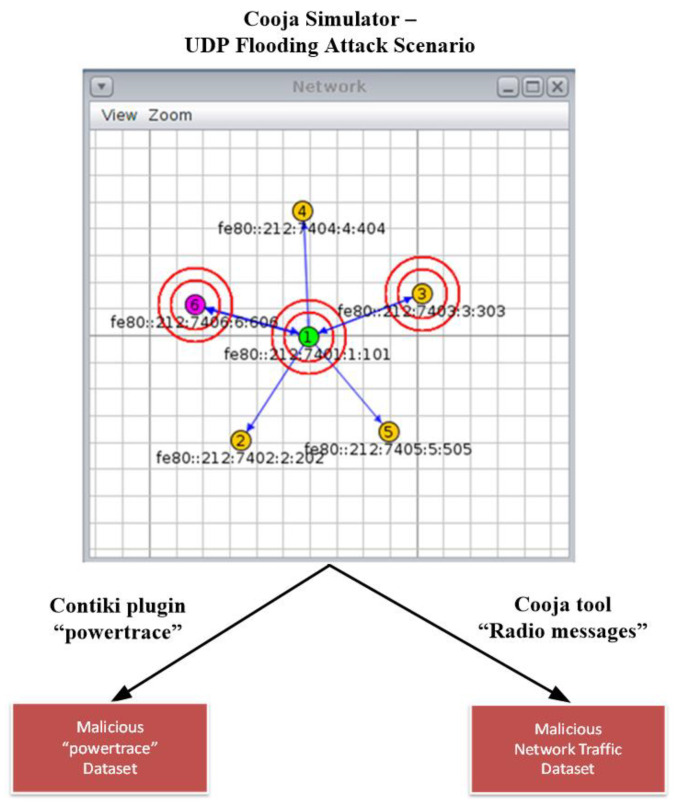
Malicious datasets generation by utilizing the Cooja simulator.

In this Section, we provide a detailed description of the approach followed to generate a set of malicious datasets by implementing a UDP flooding attack scenario in the Cooja simulator, as shown in Figure 24. Similar to the approach followed for the generation of the benign datasets in Section 4, the generated IoT/IIoT-specific information from the simulated attack scenario was captured from the Contiki plugin “powertrace” (i.e., features such as CPU consumption) and the Cooja tool “Radio messages” (i.e., network traffic features) in order to generate the “powertrace” dataset and the network traffic dataset for the simulated UDP flooding attack scenario.

**Figure 25:**
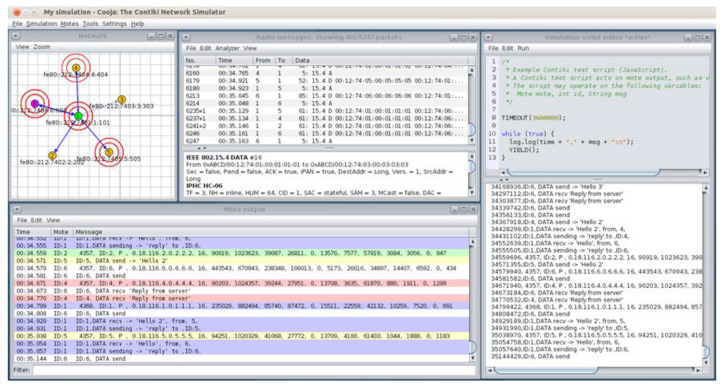
Cooja Simulator—motes’ outputs.

The network topology of the simulated UDP flooding attack scenario in the Cooja simulator environment consists of 4 yellow (benign) UDP-client motes (i.e., motes 2, 3, 4 and 5), the violet (malicious) UDP-client mote (i.e., mote 6) and the green (benign) UDP-sever mote (i.e., mote 1), as depicted in Figure 24. The simulation duration was set to 60 min and the motes’ outputs were printed out in the respective window (e.g., Mote output) while simulations run, as shown in Figure 25. Moreover, the 4 yellow (benign) UDP-client motes were configured to send text messages every 10 s, approximately, to the UDP-sever mote that was configured to provide a corresponding response. On the other hand, the violet (malicious) UDP-client mote (i.e., mote 6) was compromised with malicious code in order to send UDP packets within a very short period of time (i.e., every 200 ms). Finally, it is noteworthy to say that similar to the benign network scenario, the UDP protocol was used at the Transport Layer, the IPv6 at the network layer, and the type of motes was the Tmote Sky in the UDP flooding attack scenario.

### 5.1. Malicious “Powertrace” Dataset Generation 

#### 5.1.1. Malicious “Powertrace” Dataset Generation

**Figure 26:**
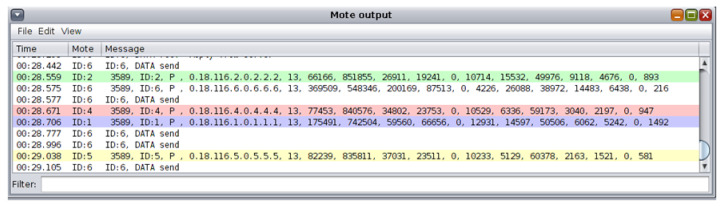
Cooja Simulator—Mote output window.

The approach followed for the “powertrace” dataset generation from the UDP flooding attack scenario was similar to the approach followed for the “powertrace” dataset generation from the benign IIoT network scenario in Section 4.1.1. In addition, the “powertrace” plugin was similarly enabled for collecting “powertrace” related features, summarised in Table 1, from the motes of the attack scenario every two seconds. In Figure 26, the depicted mote output window displays the captured “powertrace” information every two seconds and also the messages sent and received by each mote during the simulation time (60 min).

**Figure 27:**
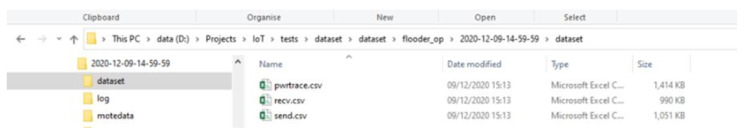
Location of the generated “pwrtrace.csv”, “recv.csv”, and “send.csv” files by the “IoT_Simul.sh” bash file.

When the timeout occurred, the simulation stopped, and all the captured information and prints were stored in the “COOJA.testlog” file. Afterwards, the “IoT_Simul.sh” file, described in Section 4.1.1, created (a) a new root folder named as “2020-12-09-14-59-59”, and (b) the “log” folder, inside the “2020-12-09-14-59-59” folder, where the “COOJA.testlog” file was copied from the “…/cooja/build” folder located in the Cooja Simulator. Then, the “IoT_Simul.sh” file following the same process, as described in Section 4.1.1, extracted the required “powertrace” information from the “COOJA.testlog” file and saved it in the “pwrtrace.csv” file in the “dataset” folder that was created by the batch file inside the “2020-12-09-14-59-59” folder, as shown below in the left part of Figure 27. In the “dataset” folder, apart from the “pwrtrace.csv” file, the “IoT_Simul.sh” file generated two more files (i.e., the “recv.csv” file and the “send.csv”), following the same process as in Section 4.1.1. The “recv.csv” file and the “send.csv” file include the “received” and “sent” messages printed by the motes, respectively.

Finally, similar to the benign “powertrace” dataset generation approach in Section 4.1.1, the “IoT_Simul.sh” file extracted the information related to each mote from the “pwrtrace.csv” file and generated one csv file for each mote with the corresponding information from the “pwrtrace.csv” file. The generated six csv files (i.e., mote1.csv, mote2.csv, mote3.csv, mote4.csv, mote5.csv, and mote6.csv) were stored in the “motedata” folder, created also by the “IoT_Simul.sh” file, as shown in the left part of Figure 27.

#### 5.1.2. Malicious “powertrace” Datasets—Results

**Figure 28:**
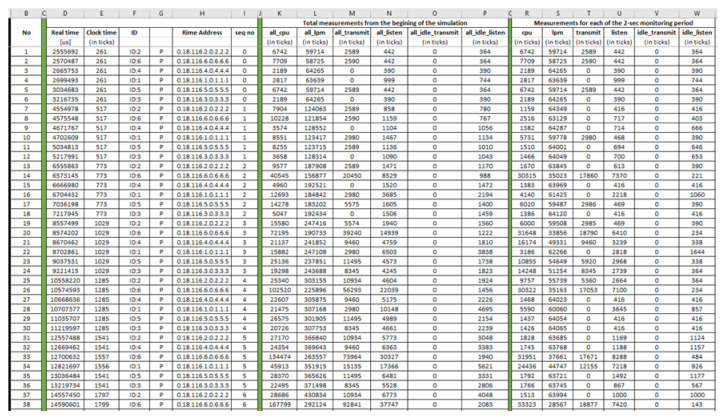
Malicious “pwrtrace.csv”—1 to 38 records.

**Figure 29:**
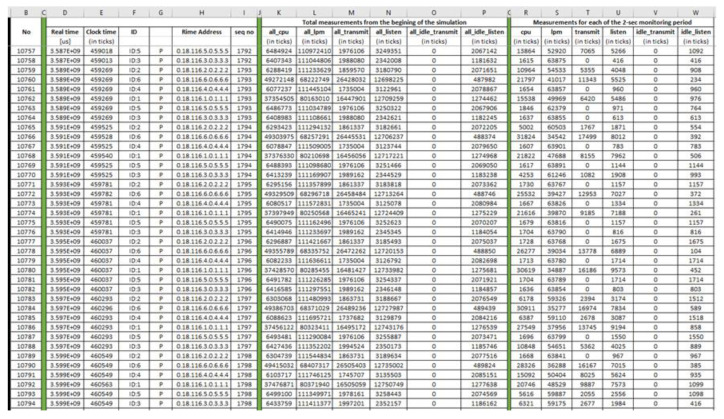
Malicious “pwrtrace.csv”—10,757 to 10,794 records.

Malicious “pwrtrace.csv”: The generated malicious “pwrtrace.csv” file consists of 10,794 records and its first 38 records (i.e., 1–38) and its last 38 records (10,757–10,794) are depicted in Figure 28 and Figure 29, respectively.

**Figure 30:**
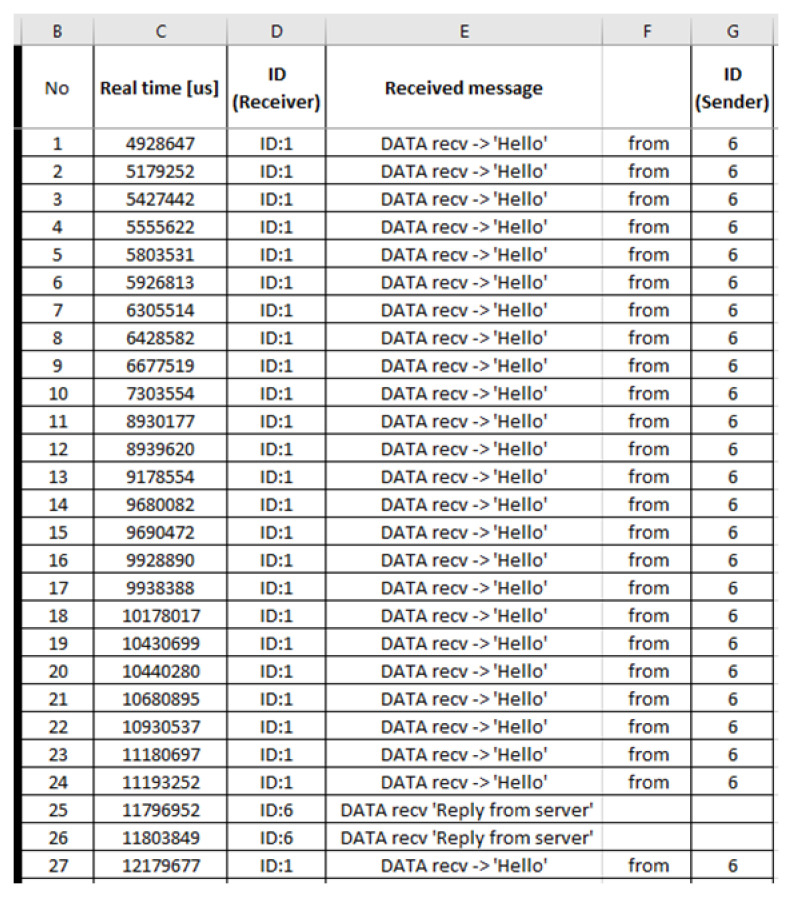
Malicious “recv.csv”—1 to 27 records.

Malicious “recv.csv”: The generated malicious “recv.csv” file consists of 21,573 records and its first 27 records (i.e., 1–27) are depicted below in Figure 30.

### 5.2. Malicious Network Traffic Dataset Generation

#### 5.2.1. Malicious Network Traffic Dataset Generation

**Figure 31:**
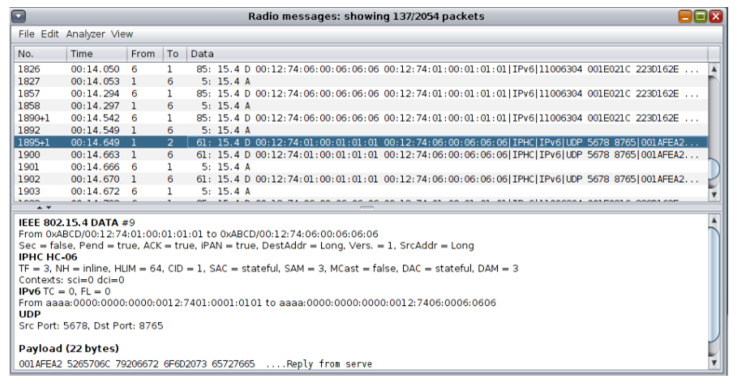
Network traffic information from the attack scenario in the “Radio messages” output window.

The approach followed for the network traffic dataset generation from the UDP flooding attack scenario was similar to the approach followed for the network traffic dataset generation from the benign IIoT network scenario in Section 4.2.1. The “Radio messages” tool, provided by the Cooja simulator, was similarly used for collecting data related to the corresponding network traffic features (e.g., source/destination IPv6 address, packet size, and communication protocol) from the network of the attack scenario. During the simulation, the network traffic information was being shown in the top part of the “Radio messages” output window as depicted in the top part of Figure 31.

**Figure 32:**
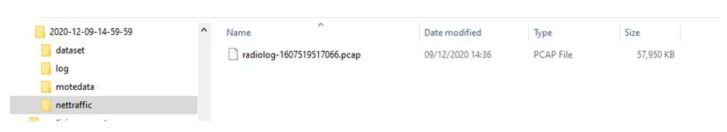
The “nettraffic” folder inside the root folder “2020-12-09-14-59-59” and the copy of the “radiolog-1607519517066.pcap” file.

When the simulation stopped, the generated pcap file was saved as “radiolog-1607519517066.pcap” within the “…/cooja/build” folder. Afterwards, the “IoT_Simul.sh” file, described in Section 4.2.1, created (a) a new root folder named as “2020-12-09-14-59-59”, and (b) the “nettraffic” folder, inside the “2020-12-09-14-59-59” folder, where the “radiolog-1607519517066.pcap” file was copied from the “…/cooja/build” folder located in the Cooja Simulator. The “nettraffic” folder inside the root folder “2020-12-09-14-59-59” and the copy of the “radiolog-1607519517066.pcap” file in the “nettraffic” folder are shown in Figure 32.

**Figure 33:**
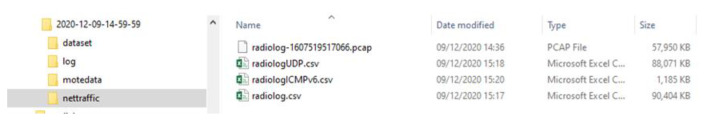
The “nettraffic” folder inside the root folder “2020-12-09-14-59-59” and its included files.

Then, following the same process, as described in Section 4.2.1, we used Wireshark to extract the stored network traffic information from the “radiolog-1607519517066.pcap” file to the “radiolog.csv” file stored in the “nettraffic” folder as shown in Figure 33.

In the “nettraffic” folder, apart from the “radiolog.csv” file, we also used Wireshark, following the same process as in Section 4.2.1, to generate two more files (i.e., the “radiologICMPv6.csv” file and the “radiologUDP.csv” file) from the “radiolog-1607519517066.pcap” file.

#### 5.2.2. Malicious Network Traffic Datasets—Results

**Figure 34:**
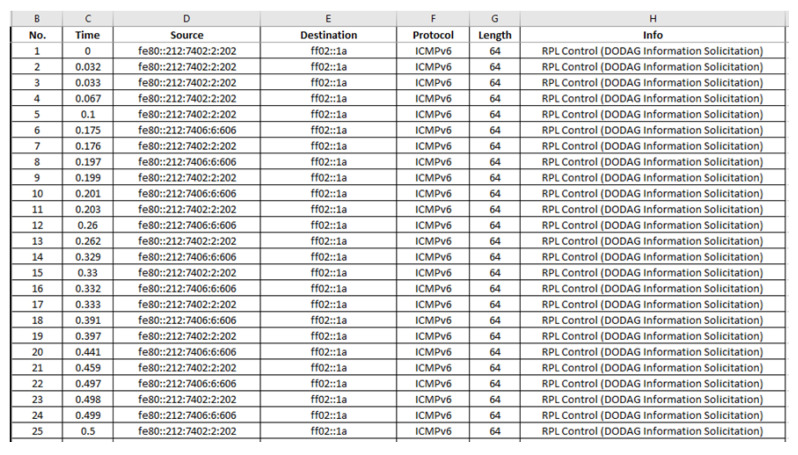
Malicious “radiolog.csv”—1 to 25 records.

“radiolog.csv”: The generated malicious “radiolog.csv” file consists of 702,332 records and its first 25 records (i.e., 1–25) are depicted below in Figure 34.

**Figure 35:**
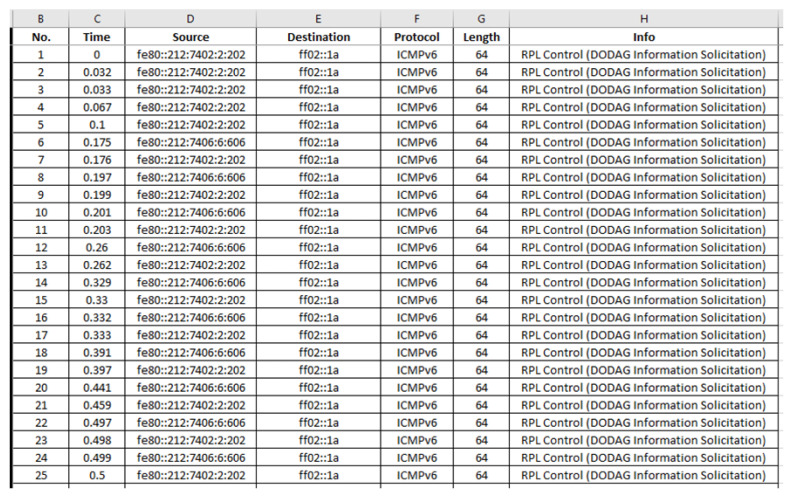
Malicious “radiologICMPv6.csv”—1 to 25 records.

“radiologICMPv6.csv”: The generated malicious “radiologICMPv6.csv” file consists of 9908 records and its first 25 records (i.e., 1–25) are depicted below in Figure 35.

**Figure 36:**
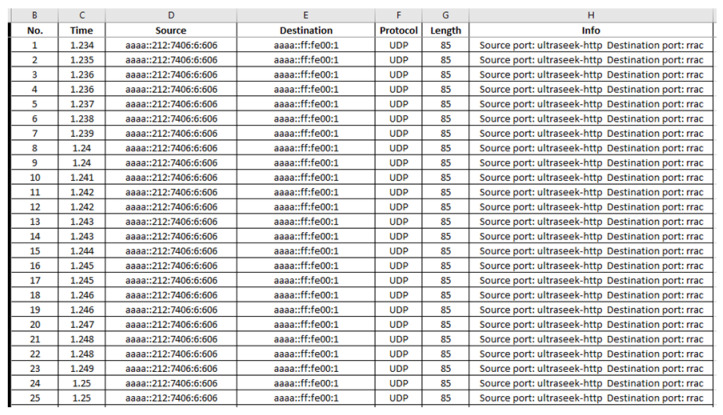
Malicious “radiologUDP.csv”—1 to 25 records.

“radiologUDP.csv”: The generated malicious “radiologUDP.csv” file consists of 670,671 records and its first 25 records (i.e., 1–25) are depicted below in Figure 36.

## 6. Discussion on the Generated Datasets 

The generated benign and malicious “pwrtrace” datasets, presented in Section 4.1.2 and Section 5.1.2, respectively, include information about raw features (e.g., all_cpu, all_lpm, all_transmit, all_listen) which can be used to derive new features more informative, in terms of the behaviour of each mote, and non-redundant. These new features are intended to constitute valuable features for training and evaluating AIDS for IoT/IIoT networks. Towards this direction, the total energy consumption of a mote in an IoT/IIoT network can be considered as a valuable feature for detection of a UDP flooding attack and its source as the compromised mote carrying out the attack is characterised by high total energy consumption, as demonstrated below. 

Based on [29,30], the total energy consumption of each mote, at the reading (i.e., record) *i*, is given by the sum of (a) the energy consumption in the CPU state; (b) the energy consumption in the LPM state; (c) the energy consumption in the Tx state; and the average power consumption Listen state, at the reading (i.e., record) *i*, as shown in the equation below:

(1) $$\begin{array}{cc} E_{{\mathrm{total}}_{i}}\left(\mathrm{mj}\right) & ={\;E}_{{\mathrm{cpu}}_{{\mathrm{total}}_{i}}}+E_{{\mathrm{lpm}}_{{\mathrm{total}}_{i}}}+E_{{\mathrm{tx}}_{{\mathrm{total}}_{i}}}+E_{{\mathrm{rx}}_{{\mathrm{total}}_{i}}}=\\ & =\left(I_{\mathrm{cpu}}\;\times V_{\mathrm{cpu}}\times T_{{\mathrm{cpu}}_{i}}\right)+\left(I_{\mathrm{lpm}}\;\times V_{\mathrm{lpm}}\;\times T_{{\mathrm{lpm}}_{i}}\right)+\left(I_{\mathrm{tx}}\times V_{\mathrm{tx}}\times T_{{\mathrm{tx}}_{i}}\right)+\left(I_{\mathrm{rx}}\times V_{\mathrm{rx}}\times T_{{\mathrm{rx}}_{i}}\right)\;\;\;\;\; \end{array}$$

where

I_cpu_: the nominal current in the CPU state;
I_lpm_: the nominal current in the LPM state;
I_tx_: the nominal current in the TX state;
I_rx_: the nominal current in the RX state;
V_cpu_: the nominal voltage in the CPU state;
V_lpm_: the nominal voltage in the LPM state;
V_tx_: the nominal voltage in the TX state;
V_rx_: the nominal voltage in the RX state;
$T_{cpu_{i}}=\;\frac{cpu_{i}\;\left(\#\;ticks\right)}{RTIMER\_ARCH\_SECOND}=\frac{cpu_{i}\;\left(\#\;ticks\right)}{32,768\;}$
$T_{lpm_{i}}=\;\frac{lpm_{i}\;\left(\#\;ticks\right)}{RTIMER\_ARCH\_SECOND}=\frac{lpm_{i}\;\left(\#\;ticks\right)}{32,768\;}$
$T_{tx_{i}}=\;\frac{tx_{i}\;\left(\#\;ticks\right)}{RTIMER\_ARCH\_SECOND}=\frac{tx_{i}\left(\#\;ticks\right)}{32,768\;}$
$T_{rx_{i}}=\;\frac{rx{\;}_{i}\left(\#\;ticks\right)}{RTIMER\_ARCH\_SECOND}=\frac{rx_{i}\;\left(\#\;ticks\right)}{32,768\;}$

Based on Equation (1) and Table 2 that provides the typical operating conditions for a Tmote Sky mote, the total energy consumption, at the reading (i.e., record) i, is given by Equation (2):

(2) $$\begin{array}{cc} E_{{\mathrm{total}}_{i}}\left(\mathrm{mj}\right) & =\;1.8\;\times 3\times \;\left(\frac{{\mathrm{cpu}}_{i}\;\left(\#\;\mathrm{ticks}\right)}{32,768\;}\right)\\ & \;+\;0.0545\;\times 3\;\times \;\left(\frac{{\mathrm{lpm}}_{i}\;\left(\#\;\mathrm{ticks}\right)}{32,768\;}\right)\;\\ & \;+19.5\;\times 3\;\times \left(\frac{{\mathrm{tx}}_{i}\left(\#\;\mathrm{ticks}\right)}{32,768\;}\right)\\ & \;+\;21.8\;\times 3\times \left(\frac{{\mathrm{rx}}_{i}\;\left(\#\;\mathrm{ticks}\right)}{32,768\;}\right)\;\;\;\;\; \end{array}$$

**Figure 37:**
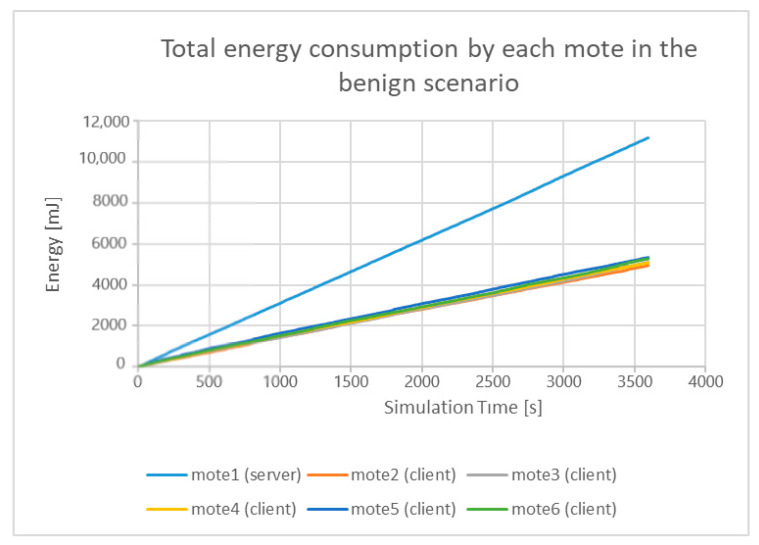
Total energy consumption by each mote in the benign scenario.

Based on Equation (2) and the following features, from the generated benign “powertrace” dataset, for each mote: (a) all_cpu; (b) all_lpm; (c) all_transmit; and (d) all_listen, the total energy consumption by each mote, during the simulation time (i.e., 60 min = 3600 s) is shown below in Figure 37.

On the other hand, based on Equation (2) and the same features (i.e., all_cpu, all_lpm, all_transmit; and all_listen) for each mote, from the generated malicious “powertrace” dataset, the total energy consumption by each mote, during the simulation time (i.e., 60 min = 3600 s) is shown below.

**Figure 38:**
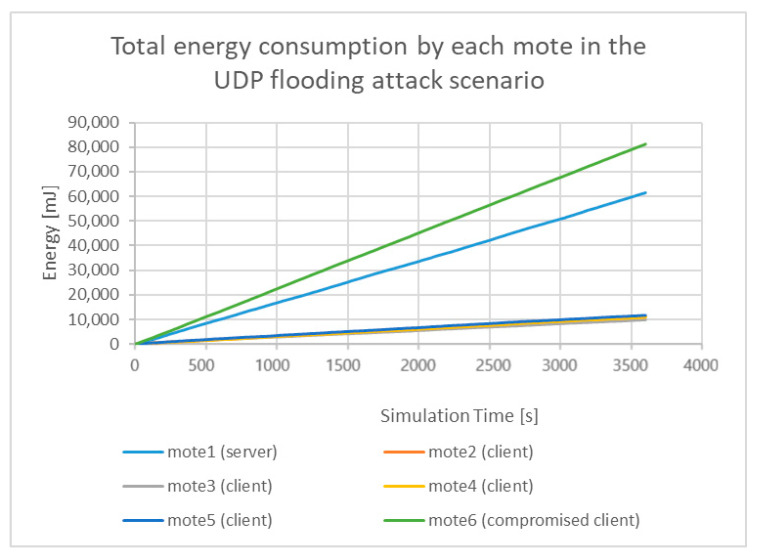
Total energy consumption by each mote in the UDP flooding attack scenario.

As shown in Figure 38, mote6, which is the compromised client that carried out the UDP flooding attack, consumed much more energy than any other legitimate client and the legitimate server in the UDP flooding attack scenario. Moreover, mote6 in the UDP flooding attack consumed much more energy than the energy it consumed in the benign scenario as demonstrated in Figure 37. 

Furthermore, the generated benign and malicious network traffic datasets, presented in Section 4.2.2 and Section 5.2.2, respectively, include information about raw features, such as source/destination address, protocol, which can be used to derive new features more informative, in terms of the behaviour of the network traffic, and non-redundant. These new features are also intended to constitute valuable features for training and evaluating AIDS for IoT/IIoT networks. From the network traffic point of view, the total RPL (Routing Protocol for Low-Power and Lossy Networks) messages overhead of the IoT/IIoT network can be considered as a feature for detection of a UDP flooding attack as an IoT/IIoT network under a UDP flooding attack is characterised by low total RPL messages overhead because of the huge amount of the UDP messages flooding the network, as shown below.

Table 3 was extracted from the benign network traffic dataset (i.e., benign “radiolog.csv”) and shows, in the last column, the percentage of the RPL messages overhead per mote which is calculated as follows: the number of RPL messages per mote over the total number of exchanged messages within the network during the simulation time (i.e., 116,463 messages). The last row of Table 3 contains the total number of RPL messages (7975), UDP messages (104,048), and other protocol messages (4440) exchanged within the network, and the total RPL messages overhead (%).

**Figure 39:**
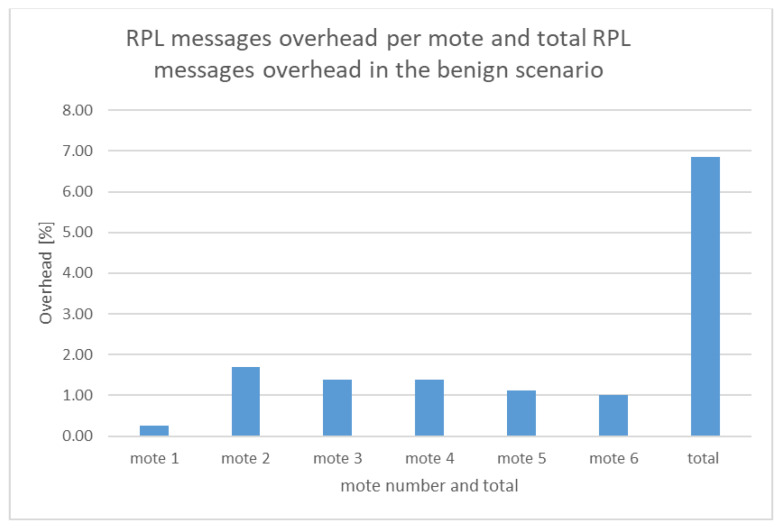
RPL messages overhead per mote and total RPL messages overhead in the benign scenario.

Based on the information included in Table 3, the calculated RPL messages overhead per mote and the total RPL messages overhead are depicted in Figure 39.

On the other hand, Table 4 was extracted from the malicious network traffic dataset (i.e., malicious “radiolog.csv”) reflecting the UDP flooding attack scenario. Similar to Table 3, Table 4 shows, in the last column, the percentage of the RPL messages overhead per mote which is calculated as follows: the number of RPL messages per mote over the total number of exchanged messages within the network during the simulation time (i.e., 702,332 messages). The last row of Table 4 contains the total number of RPL messages (9908), UDP messages (670,671), and other protocol messages (21,753) exchanged within the network, and the total RPL messages overhead (%).

**Figure 40:**
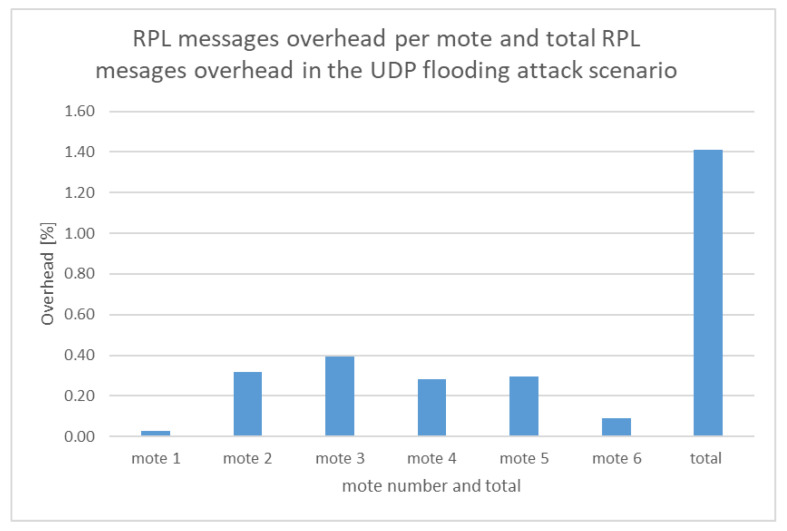
RPL messages overhead per mote and total RPL messages overhead in the malicious scenario.

Based on the information included in Table 4, the calculated RPL messages overhead per mote and the total RPL messages overhead are depicted in Figure 40.

As shown in Figure 39 and Figure 40, the total RPL messages overhead (1.41%) in the malicious scenario is much less than the total RPL messages overhead in the benign scenario (6.85%) because of the huge amount of the UDP messages flooding the network in the malicious scenario.

## 7. Conclusions

Due to the urgent need for up-to-date, representative and well-structured IoT/IIoT-specific datasets which are publicly available and constitute benchmark datasets for training and evaluating ML models used in AIDSs for IoT/IIoT networks, we target the generation of new labelled IoT/IIoT datasets that will be publicly available to the research community and include (i) events reflecting multiple benign and attack scenarios from current IoT/IIoT network environments, (ii) sensor measurement data, (iii) network-related information (e.g., packet-level information and flow-level information) from the IoT/IIoT network, and (iv) information related to the behaviour of the IoT/IIoT devices deployed within the IoT/IIoT network. In this context, this paper we presented an initial set of datasets with these significant characteristics for effective training and testing of ML models used in AIDSs for protecting IoT/IIoT networks. In particular, the provided set of datasets consists of (a) benign IoT/IIoT datasets (i.e., around 11,000 records of the benign “powertrace” dataset and around 116,000 records of the benign network traffic dataset), and (b) malicious IoT/IIoT datasets (i.e., around 11,000 records of the malicious “powertrace” dataset and around 700,000 records of the malicious network traffic dataset).

In addition, in this paper, we presented in detail the approach that we adopted to generate the initial set of benign IoT/IIoT and malicious IoT/IIoT datasets by utilising the Cooja simulator that was the simulation environment where the corresponding benign and attack scenarios were implemented. It is worthwhile to highlight that for the first time and to the best of our knowledge, that the Cooja simulator, which is the companion network simulator of Contiki OS (one of the most popular OSs for resource constrained IoT devices), was used in a systematic way in order to generate IoT/IIoT datasets. In particular, we provided a comprehensive description of the whole approach we followed in order to acquire the generated datasets within csv files from the captured raw information residing in the Cooja simulator environment. Then, the generated datasets in csv format are ready to feed ML algorithms for training and testing purposes.

Our goal is that the new labelled IoT/IIoT datasets generated by utilizing the Cooja simulator should not to be considered as a replacement of datasets captured from real IoT/IIoT networks or real IoT/IIoT testbeds, but instead to be considered as complementary datasets that will contribute to fill the gap in the lack of publicly available up-to-date, representative and well-structured IoT/IIoT-specific datasets that constitute benchmark datasets for training and evaluating ML models used in AIDSs for IoT/IIoT networks.

As future work, we plan to continue working on the implementation of more benign IoT/IIoT network scenarios and various types of IoT/IIoT network attack scenarios, with more motes, in Cooja simulator in order to generate richer benign and malicious datasets for more effective training and testing of ML algorithms used in AIDSs for protecting IoT/IIoT networks such as the one described in [31]. Our intention is to make the generated rich datasets publicly available to the research community. In addition, we will also make publicly available the Cooja-based framework that will have been developed in order to generate the rich datasets. This will allow researchers to reproduce datasets as well as generate new datasets for their own scenarios without having to “reinvent the wheel”. Furthermore, we intend to analyse the generated datasets to select the most appropriate features for accurate and efficient detection of different types of attacks within an IoT/IIoT network. Finally, we plan to apply a number of common ML algorithms (e.g., support vector machines (SVMs), Naïve Bayes, k-nearest neighbour, logistics regression, etc.) to evaluate their performance on the new generated datasets when these algorithms are used for anomaly detection in AIDSs.

## Figures and Tables

**Table 1:** “powertrace” plugin—Set of Captured Features.

Index	Feature	Description
1	sim time	simulation time
2	clock_time()	clock time (i.e.,by default, 128 ticks/second)
3	ID	Mote ID
4	P	label
5	rimeaddr	rime address
6	seqno	sequence number
7	all_cpu	accumulated CPU energy consumption
8	all_lpm	accumulated Low Power Mode energy consumption
9	all_transmit	accumulated transmission energy consumption
10	all_listen	accumulated listen energy consumption
11	all_idle_transmit	accumulated idle transmission energy consumption
12	all_idle_listen	accumulated idle listen energy consumption
13	cpu	CPU energy consumption for this cycle
14	lpm	LPM energy consumption for this cycle
15	transmit	transmission energy consumption for this cycle
16	listen	listen energy consumption for this cycle
17	idle_transmit	idle transmission energy consumption for this cycle
18	idle_listen	idle listen energy consumption for this cycle

**Table 2:** Typical Operating Conditions for Tmote Sky motes.

	MIN	NOM (Typical)	MAX	UNIT
Supply voltage	2.1	3.0	3.6	V
Supply voltage during flash memory programming	2.7	3.0	3.6	V
Operating free air temperature	−40		85	ºC
Current Consumption: MCU on, Radio RX		21.8	23	mA
Current Consumption: MCU on, Radio TX		19.5	21	mA
Current Consumption: MCU on, Radio off		1800	2400	µA
Current Consumption: MCU idle, Radio off		54.5	1200	µA
Current Consumption: MCU standby		5.1	21.0	µA

**Table 3:** RPL messages overhead of the IoT/IIoT network in the benign scenario.

RPL Messages Overhead				
	Number of RPL Messages	Number of UDP Messages	Number of Other Messages	RPL Overhead (%)
Mote 1	290	43,804	N/A	0.25
Mote 2	1982	11,621	N/A	1.70
Mote 3	1621	11,883	N/A	1.39
Mote 4	1604	11,827	N/A	1.38
Mote 5	1308	12,556	N/A	1.12
Mote 6	1170	12,357	N/A	1.00
Total	7975	104,048	4440	6.85

**Table 4:** RPL messages overhead of the IoT/IIoT network in the benign scenario.

RPL Messages Overhead				
	Number of RPL Messages	Number of UDP Messages	Number of Other Messages	RPL Overhead (%)
Mote 1	203	254,796	N/A	0.03
Mote 2	2228	28,953	N/A	0.32
Mote 3	2768	30,238	N/A	0.39
Mote 4	1976	27,260	N/A	0.28
Mote 5	2084	31,247	N/A	0.30
Mote 6	6490	298,177	N/A	0.09
Total	9908	670,671	21,753	1.41
